# Improved Operation of the Modified Non-Inverting Step-Down/Up (MNI-SDU) DC-DC Converter

**DOI:** 10.3390/mi16091063

**Published:** 2025-09-20

**Authors:** Juan A. Villanueva-Loredo, Julio C. Rosas-Caro, Panfilo R. Martinez-Rodriguez, Christopher J. Rodriguez-Cortes, Diego Langarica-Cordoba, Gerardo Vazquez-Guzman

**Affiliations:** 1 School of Sciences, Universidad Autónoma de San Luis Potosí (UASLP), San Luis Potosí 78295, San Luis Potosí, Mexico; juan.villanueva@uaslp.mx (J.A.V.-L.); a369184@alumnos.uaslp.mx (C.J.R.-C.); diego.langarica@uaslp.mx (D.L.-C.); gerardo.guzman@uaslp.mx (G.V.-G.); 2 Facultad de Ingenieria, Universidad Panamericana, Zapopan 45010, Jalisco, Mexico

**Keywords:** PWM converter, voltage regulator, buck-boost converter, battery voltage regulation, non-pulsating input current, two-loop control, asynchronous duty-cycle control

## Abstract

This paper presents an enhanced operation strategy for a recently proposed converter called Modified Non-Inverting Step-Down/Up (MNI-SDU) DC-DC converter intended for battery voltage regulation. Unlike the conventional approach, where both switching stages share a single duty cycle, the proposed method controls asynchronously the two duty cycles through a fixed time offset to optimize performance. A methodology is developed to define suitable duty cycle ranges that ensure proper converter operation according to input/output voltage specifications, while simultaneously reducing the current and voltage ripples and electrical stress in the capacitor and semiconductors. Furthermore, a model-based control strategy is proposed, taking into account the enhanced operational characteristics. Consequently, a PI-PI current-mode controller is designed using loop shaping techniques to maintain the output voltage regulated at the desired level. The proposed approach is analyzed mathematically and validated through experimental results. The findings demonstrate that optimizing through asynchronous duty-cycle control with a fixed time offset improves ripple, stress values, and overall efficiency, while maintaining robust output voltage regulation, making this method well-suited for applications requiring compact and reliable power conversion.

## 1. Introduction

Power electronics is a field of electrical engineering that focuses on the efficient conversion, control, and conditioning of electrical energy using semiconductor-based switching devices [1,2]. In the modern world, particularly in renewable energy applications, it plays a vital role by enabling the interface between different voltage levels, loads, and storage elements with minimal losses [3,4]. The power conversion units, usually called converters, have a wide range of applications, not only renewable energy systems, but electric vehicles, industrial motor drives, telecommunications, and aerospace systems [5,6]. As the demand for energy-efficient and high-performance power conversion continues to grow, the development of optimized converter topologies and control strategies becomes increasingly relevant [6,7].

Power conversion can be classified into different types based on the nature of the input and output signals. DC-DC conversion involves the transformation of one direct current (DC) voltage level to another, either increasing (boost), decreasing (buck), or both (buck-boost) while maintaining efficiency and control. DC-AC conversion, also known as inversion, converts direct current into alternating current (AC) with a specified frequency and waveform, allowing DC sources to power AC loads. AC-DC conversion, or rectification, is the process of converting alternating current into direct current, typically for powering electronic circuits and devices. Finally, AC-AC conversion modifies the characteristics of an AC signal, such as its voltage, frequency, or phase, to suit different applications and improve power quality [1,2].

Among DC-DC conversion applications, there are scenarios where a buck-boost converter is required to manage input voltage variations while maintaining a stable output. Buck-Boost converters are essential in systems where the power source exhibits voltage variations around its nominal level [8,9]. Notable applications include energy systems and transportation platforms powered by fuel cells, battery-operated electric vehicles, and more electric aircraft (MEA). Additionally, they are essential in portable telecommunications equipment. In all these cases, a power interface is necessary to stabilize the supply voltage and ensure reliable operation of the connected loads [10,11,12].

One of the most critical applications is battery voltage regulation, where the voltage level can fluctuate due to charge and discharge cycles. This is particularly relevant in systems powered by lithium-ion batteries, fuel cells, and supercapacitors, which require efficient voltage adaptation to ensure proper operation [13,14,15,16]. Furthermore, for these particular sources, it is recommended to avoid periodic pulse current patterns during discharge, as these sudden fluctuations can be harmful [14]. Therefore, the converter used to regulate the voltage of these sources needs to have a smooth, non-pulsating input current, meaning the current does not change suddenly from one level to another. For this current to be as constant as possible, it must be ripple-free or have a small ripple value.

There is a variety of buck-boost DC-DC converters with low current ripples and high efficiency, along with control schemes that are suitable to regulate the voltage fluctuation of a source. In [17], an n-cell interleaved buck-boost with a non-linear adaptive controller is proposed to regulate the voltage of a fuel cell. Here, the current ripple can be reduced n-times, ensuring a substantial increase in power density. In [18], a buck–boost converter with high efficiency and small ripple content applied to extend the battery life of portable devices is proposed. It uses a hybrid feedforward technique to control four active switches, which minimizes the switching and conduction losses. In [19], a buck-boost converter with low input current for PEM fuel cell applications is proposed. It features low components and a current-mode control scheme for output voltage regulation. In [20], a quadratic buck–boost converter with non-pulsating input current suitable for renewable energy applications is proposed. It uses a PI controller to provide output voltage regulation with output voltage ripple cancellation.

**Figure 1:**
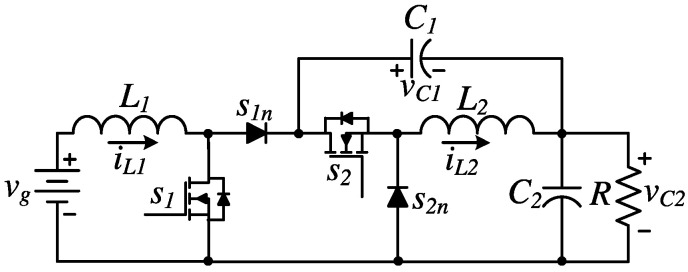
The topology of the MNI-SDU converter.

One of the recent contributions to buck-boost DC-DC conversion was introduced in [16] as a non-inverting topology specifically designed to efficiently regulate voltage in scenarios where the input can fluctuate above or below the target output. Figure 1 shows the discussed topology, referred to in this article as the MNI-SDU converter (Modified Non-Inverting Step Down/Up). This converter employs a non-cascaded interconnection of boost and buck-boost stages, enabling partial direct power transfer from input to output, thereby reducing redundant power processing and improving overall efficiency. Unlike conventional buck-boost designs, this topology delivers a non-inverted output voltage, which is crucial for systems that require consistent polarities, such as embedded electronics and battery-powered devices. In addition, the converter maintains a continuous input current, which helps significantly reduce input ripple. This characteristic is especially important when working with sensitive energy sources such as lithium-ion batteries or fuel cells. The converter’s original control strategy, based on a single duty cycle driving both stages simultaneously, provides stable output regulation across a wide input range, making it a reliable solution for modern power management applications.

This article proposes an enhanced operational strategy for the aforementioned non-inverting step-down/up DC-DC converter by asynchronously controlling the two active switches with a fixed time offset. This operation enables an additional switching state for power flow and voltage gain distribution between stages. This approach aims to reduce current and voltage ripples and minimize voltage stress on passive components. A methodology is developed to select the fixed time offset defining the suitable operating regions for each duty cycle according to the desired input/output voltage ratio, ensuring proper operation across the full regulation range. Mathematical modeling of the MNI-SDU, and a PI-PI current-mode control design based on loop shaping techniques are provided. Experimental results of a 570 W prototype validate the effectiveness of the proposed strategy and demonstrate its benefits in terms of performance, component ripple, voltage stress reduction, and output voltage regulation. Hence, the main contributions are the following:

1. An asynchronous operation mode with a fixed time offset for the MNI-SDU converter, which reduces ripple, stress levels, and power losses.
2. Design expressions of the MNI-SDU converter in this proposed operation.
3. A methodology to select an appropriate value for the fixed time offset.
4. The linearized model and transfer functions of the MNI-SDU converter under the proposed operation mode.
5. A methodology to select adequate parameters of a current-mode controller.
6. An efficiency analysis demonstrating enhanced efficiency.
7. Experimental results validating the theoretical analysis.

The outline of the paper is as follows. Section 2 describes the original operation of the MNI-SDU converter. Section 3 introduces the proposed improved operation of the converter. Section 4 presents the linearized model of the converter, the transfer functions, and the methodology for selecting appropriate parameters of a PI-PI current mode controller. Section 5 details the prototype design and experimental graphs for the validation of the system. Finally, Section 6 provides conclusions and remarks.

## 2. The MNI-SDU Converter in Its Original Operation

**Figure 2:**
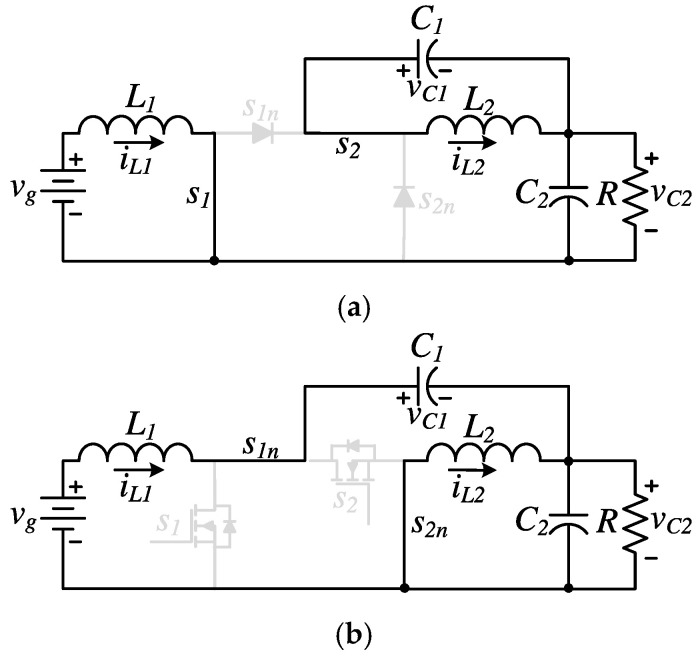
Equivalent circuits according to the switching state in the synchronized operation: (**a**) ON, (**b**) OFF.

The original operation of the MNI-SDU converter proposed in [16] is based on the synchronized switching of both active transistors. In this configuration, the two switches operate with a common duty cycle and are turned ON and OFF simultaneously during each switching period. This synchronized control results in two equivalent circuits in continuous conduction mode (CCM), as described in [16]. These equivalent circuits correspond to the state in which transistors are closed while diodes are off (see Figure 2a), and then transistors are off while diodes are closed (see Figure 2b).

From the equivalent circuits and by applying the standard averaging technique, the average dynamic behavior of the converter can be described by the following set of differential equations,

(1) $$L_{1}\frac{di_{L1}}{dt}=dv_{g}+\left(1-d\right)\left(v_{g}-v_{C1}-v_{C2}\right),$$

(2) $$L_{2}\frac{di_{L2}}{dt}=dv_{C1}+\left(1-d\right)\left(-v_{C2}\right),$$

(3) $$C_{1}\frac{dv_{C1}}{dt}=d\left(-i_{L2}\right)+\left(1-d\right)i_{L1},$$

(4) $$C_{2}\frac{dv_{C2}}{dt}=d\left(-i_{o}\right)+\left(1-d\right)\left(i_{L1}+i_{L2}-i_{o}\right),$$

where $i_{L1}$ and $i_{L2}$ are the current of the inductors $L_{1}$ and $L_{2}$; while $v_{C1}$ and $v_{C2}$ are the voltage of the capacitors $C_{1}$ and $C_{2}$, respectively. The input voltage is represented by $v_{g}$, the output current by $i_{o}$, and the duty cycle by d. Equations (1)–(4) can be algebraically simplified to

(5) $$L_{1}\frac{di_{L1}}{dt}=v_{g}-\left(1-d\right)\left(v_{C1}+v_{C2}\right),$$

(6) $$L_{2}\frac{di_{L2}}{dt}=dv_{C1}-\left(1-d\right)v_{C2},$$

(7) $$C_{1}\frac{dv_{C1}}{dt}=\left(1-d\right)i_{L1}-di_{L2},$$

(8) $$C_{2}\frac{dv_{C2}}{dt}=\left(1-d\right)\left(i_{L1}+i_{L2}\right)-i_{o}.$$

Based on the dynamic Equations (5)–(8), the steady-state operation of the converter can be analyzed by setting the time derivatives to zero. This corresponds to the converter operating in equilibrium, where average voltages and currents remain constant over time. Under this condition, the equilibrium equations can be written as Equations (9)–(12). To clearly distinguish the steady-state values from their time-varying counterparts, the variables in the equilibrium equations are denoted using uppercase letters as follows:

(9) $$V_{C1}=V_{g},$$

(10) $$V_{C2}=\frac{D}{1-D}V_{g},$$

(11) $$I_{L1}=\left(\frac{D}{1-D}\right)I_{o},$$

(12) $$I_{L2}=I_{o}.$$

Finally, for the design and selection of the passive components, it is important to define the switching peak-to-peak ripple specifications for inductor currents (Δ*i_L_*_1_ and Δ*i_L_*_2_) and capacitor voltages (Δ*v_C_*_1_ and Δ*v_C_*_2_). The switching period is defined by *T_S_*. Once the desired ripple values are established, the required values for inductors (*L*_1_ and *L*_2_) and capacitors (*C*_1_ and *C*_2_) can be computed using the steady-state ripple relationships.

(13) $$\Delta i_{L1}=\frac{V_{g}}{L_{1}}DT_{S},$$

(14) $$\Delta i_{L2}=\frac{V_{g}}{L_{2}}DT_{S},$$

(15) $$\Delta v_{C1}=\frac{I_{o}}{C_{1}}DT_{S},$$

(16) $$\Delta v_{C2}=\frac{I_{o}}{C_{2}}DT_{S}.$$

The inductor *L*_1_ gets charged with the input voltage source *V_g_* when the transistors are closed; at the same time, the inductor *L*_2_ gets charged with the voltage across capacitor *C*_1_, but since $V_{C1}=V_{g}$ according to Equation (9), their ripple equations are similar. Something similar happens to the capacitors; *C*_1_ gets discharged by the current through *L*_2_ when transistors are closed, and at the same time, *C*_2_ gets discharged by the output current; since *I_L_*_2_ *= I_o_*, according to Equation (12), their ripple equations are similar. The converter output voltage is *V_C_*_2_; from Equation (10), we can corroborate that the converter gain is the same gain as in the traditional step-down/up or buck-boost converter. Furthermore, an important aspect for the selection of semiconductors is their stress voltage, which is the same for all elements and is equivalent to the sum of the voltage of the capacitors, as can be seen in Figure 2. Thus, by adding the Equations (9) and (10), results in

(17) $$V_{s1}=V_{s2}=V_{s1n}=V_{s2n}=\frac{V_{g}}{1-D}.$$

## 3. The Proposed Operation

Let us now introduce the proposed operation strategy in which the two transistors of the converter are controlled asynchronously. Unlike the conventional synchronized operation, where both transistors are switched simultaneously with a common duty cycle, the proposed method assigns a duty cycle to each transistor. This decoupled switching approach increases the degrees of freedom in the converter’s operation, allowing greater flexibility in power flow distribution between stages. In particular, it enables the adjustment of the first stage’s duty cycle to minimize input current ripple, while the second stage can be optimized for voltage regulation.

**Figure 3:**
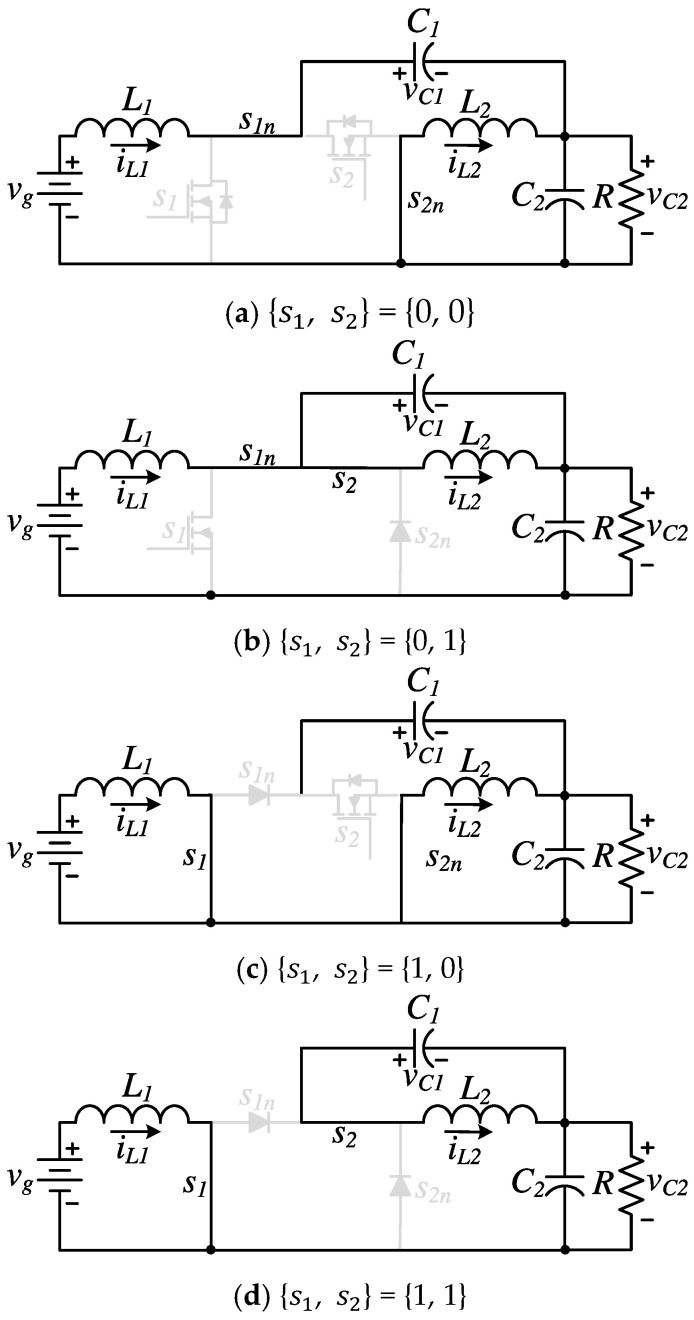
Equivalent circuits enabling independent control of the two active switches: (**a**) {0,0}, (**b**) {0,1}, (**c**) {1,0}, (**d**) {1,1}.

The following analysis describes the switching behavior, operating intervals, and resulting waveforms under this new control strategy. With independent duty cycles for the two active switches, let’s say $d_{1}$ for $s_{1}$ ($s_{1n}$ is its logical inverse) and $d_{2}$ for $s_{2}$ ($s_{2n}$ is its logical inverse), the converter can now operate in four distinct switching states, corresponding to all possible combinations of the ON/OFF status of the set {$s_{1}$, $s_{2}$}. These states are defined as {0, 0}, {0, 1}, {1, 0}, and {1, 1}, where each pair indicates the activation state of the first ($s_{1}$) and second switch ($s_{2}$), respectively. This represents a significant change from the conventional synchronized operation, which involved only two switching states per cycle. The four switching states and their associated circuit configurations are illustrated in Figure 3.

**Figure 4:**
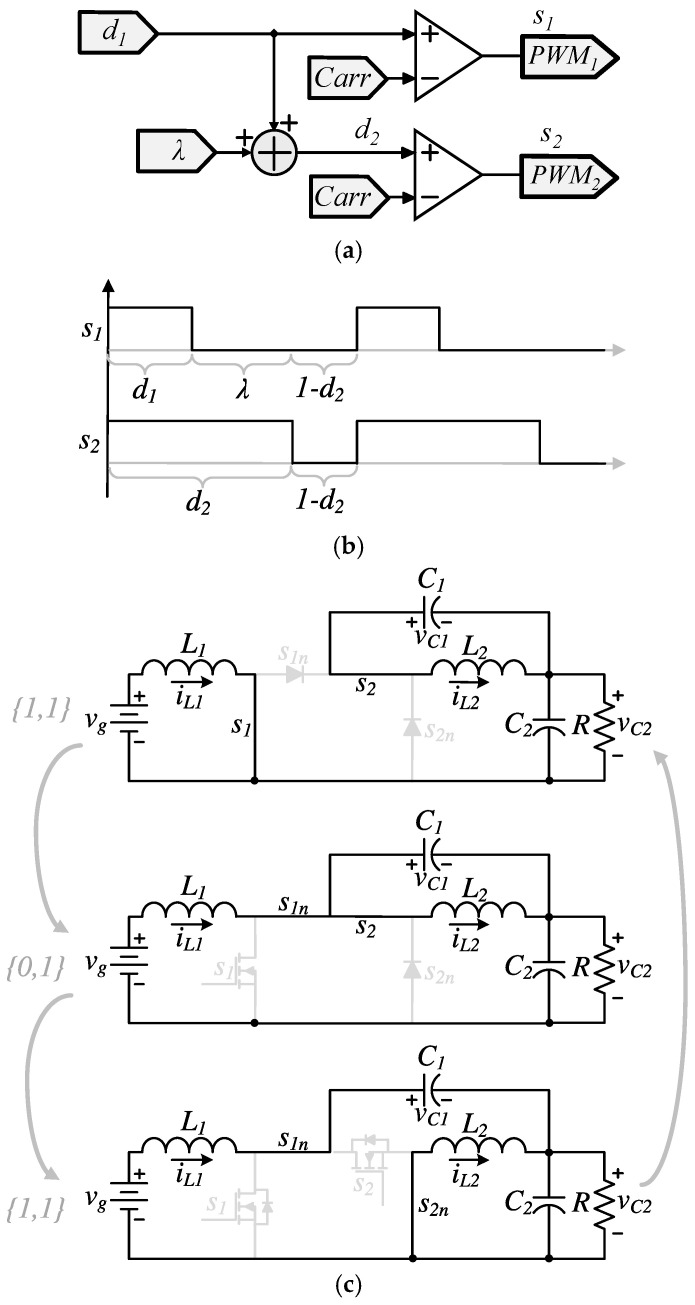
Proposed operation strategy: (**a**) relation among duty cycles, (**b**) firing signals in the proposed operation, (**c**) active switching states.

The analysis of a converter with four switching states is more complex compared with the conventional case, due to the increased number of possible transitions and operating intervals within each switching period. However, as will be demonstrated in the results section, operating the converter an asynchronous control of the two switches offers performance benefits. To simplify, a constrained relationship between the two duty cycles is proposed as follows:

(18) $$d_{2}=d_{1}+\lambda ,$$

where $d_{1}$ represents the duty cycle of the transistor $s_{1}$, $d_{2}$ the duty cycle of the transistor $s_{2}$, and *λ* a fixed constant offset between 0 and 1. Figure 4a illustrates the block diagram of this rule, and Figure 4b presents the firing signals produced by this rule. Figure 4c shows the sequence of equivalent circuits resulting from the PWM signals in Figure 4b. It can be observed that the proposed condition results in the first transistor consistently operating with a lower duty cycle than the second one. As a result, one of the four switching states {1, 0} becomes unreachable during normal operation, effectively reducing the number of active switching states from four to three.

From Figure 4, and by applying the averaging technique, the average model of the converter can be described by the following set of differential equations,

(19) $$L_{1}\frac{di_{L1}}{dt}=d_{1}v_{g}+\lambda \left(v_{g}-v_{C1}-v_{C2}\right)+\left(1-d_{2}\right)\left(v_{g}-v_{C1}-v_{C2}\right),$$

(20) $$L_{2}\frac{di_{L2}}{dt}=d_{1}v_{C1}+\lambda v_{C1}+\left(1-d_{2}\right)\left(-v_{C2}\right),$$

(21) $$C_{1}\frac{dv_{C1}}{dt}=d_{1}\left(-i_{L2}\right)+\lambda \left(i_{L1}-i_{L2}\right)+\left(1-d_{2}\right)i_{L1},$$

(22) $$C_{2}\frac{dv_{C2}}{dt}=d_{1}\left(-i_{o}\right)+\lambda \left(i_{L1}-i_{o}\right)+\left(1-d_{2}\right)\left(i_{L1}+i_{L2}-i_{o}\right).$$

By assuming $d_{2}=d_{1}+\lambda$, the system model simplifies to

(23) $$L_{1}\frac{di_{L1}}{dt}=v_{g}-\left(1-d_{1}\right)\left(v_{C1}+v_{C2}\right),$$

(24) $$L_{2}\frac{di_{L2}}{dt}=(d_{1}+\lambda )v_{C1}-\left(1-d_{1}-\lambda \right)v_{C2},$$

(25) $$C_{1}\frac{dv_{C1}}{dt}=\left(1-d_{1}\right)i_{L1}-(d_{1}+\lambda )i_{L2},$$

(26) $$C_{2}\frac{dv_{C2}}{dt}=\left(1-d_{1}\right)i_{L1}+\left(1-d_{1}-\lambda \right)i_{L2}-i_{o}.$$

Based on Equations (23)–(26), as well as in the previous operation, the converter equilibrium can be analyzed by setting the time derivatives to zero, where average voltages and currents remain constant over time. Under this condition, the equilibrium equations can be written as

(27) $$V_{C1}=\left(\frac{1-D_{1}-\lambda }{1-D_{1}}\right)V_{g},$$

(28) $$V_{C2}=\left(\frac{D_{1}+\lambda }{1-D_{1}}\right)V_{g},$$

(29) $$I_{L1}=\left(\frac{D_{1}+\lambda }{1-D_{1}}\right)I_{o},$$

(30) $$I_{L2}=I_{o}.$$

According to (28), the conversion ratio *M* results in

(31) $$M=\frac{V_{C2}}{V_{g}}=\frac{D_{1}+\lambda }{1-D_{1}}.$$

Finally, for the design and selection of the passive components, it is important to define the switching peak-to-peak ripple specifications for inductor currents (Δ*i_L_*_1_ and Δ*i_L_*_2_) and capacitor voltages (Δ*v_C_*_1_ and Δ*v_C_*_2_). Then, the required values for inductors (*L*_1_ and *L*_2_) and capacitors (*C*_1_ and *C*_2_) can be obtained using the steady-state ripple relationships as follows

(32) $$\Delta i_{L1}=\frac{V_{g}}{L_{1}}D_{1}T_{S},$$

(33) $$\Delta i_{L2}=\frac{V_{g}}{L_{2}}\left(\frac{1-D_{1}-\lambda }{1-D_{1}}\right)(D_{1}+\lambda )T_{S},$$

(34) $$\Delta v_{C1}=\frac{I_{o}}{C_{1}}D_{1}T_{S},$$

(35) $$\Delta v_{C2}=\frac{I_{o}}{C_{2}}D_{1}T_{S}.$$

As observed in the design equations, the ripples are computed using the same fundamental expression as in the conventional synchronized case. However, due to the proposed relationship between duty cycles, the value of *D*_1_ required to achieve a given voltage gain is lower. This reduction in *D*_1_ directly leads to smaller ripple values or the possibility of choosing smaller inductances and capacitances while maintaining the same ripple. This advantage becomes more evident when comparing the ripple values and performance metrics between the traditional and proposed strategies, as discussed in Section 5. For the selection of semiconductors, it is important to consider the voltage stress values. In the proposal, all semiconductors are exposed to the same value of stress voltage, i.e., $V_{s1}=V_{s2}=V_{s1n}=V_{s2n}$. This value is the sum of the voltages across the capacitors, as shown in Figure 4. Therefore, in this case, the voltage stress across the semiconductors is obtained by directly adding Equations (27) and (28), resulting in

(36) $$V_{STRESS}=V_{s1}=V_{s2}=V_{s1n}=V_{s2n}=\frac{V_{g}}{1-D_{1}}.$$

### Selection of the Fixed Time Offset λ

One of the main contributions of this paper is the asynchronous control of the two active switches through a fixed time offset represented by *λ*. The selection of the parameter *λ* plays an important role in shaping the operational behavior of the converter. Specifically, the value of *λ* influences the range of achievable voltage gains and should be chosen according to the specific requirements of the application. A smaller value allows the second stage to contribute more significantly to voltage conversion, whereas a larger value prioritizes the reduction in ripple and stress voltage. Therefore, this section presents a methodology for selecting *λ* for specific application requirements. This criterion is based on choosing the highest value of *λ* that meets the voltage gain requirements of the specific application without exceeding the critical duty cycle limits. The methodology is described in the following steps:

1. Define the specific voltage requirements according to the application: the minimum voltage of the source $V_{gmin}$, the maximum voltage of the source $V_{gmax}$, and the desired voltage reference $V_{ref}$. Remember that, in steady state $V_{C2}=V_{ref}$.
2. Obtain the maximum and minimum voltage gain according to,
  (37) $$M_{\min}=\frac{V_{C2}}{V_{g\max}},$$
  (38) $$M_{\max}=\frac{V_{C2}}{V_{g\min}}.$$
3. Determine the minimum and maximum critical duty cycles, denoted as $D_{crit-min}$ and $D_{crit-max}$, at which the converter operates reliably. To ensure proper operation of the converter, the duty cycle must satisfy the following inequality,
  (39) $$D_{crit-\min}\leq D_{1}<D_{1}+\lambda \leq D_{crit-\max}.$$
4. Obtain λ values that meet the maximum and minimum requirements. The nominal duty cycle for the active switch $s_{1}$ is $D_{1}$ and for the active switch $s_{2}$ is $D_{1}+\;\lambda$, where 0<λ<1. Thus, the possible minimum value of $D_{1}$ is $D_{crit-min,}$ and the possible maximum value of $D_{1}+\;\lambda$ is $D_{crit-max}$. According to (31), the voltage gain value $M=(D_{1}+\lambda )/(1-D_{1})$. Thus, the value of *λ* to obtain the minimum voltage gain with the critical minimum duty cycle conditions is
  (40) $$\lambda_{a}=-(1+M_{\min})D_{crit-\min}+M_{\min}.$$

Meanwhile, the value of *λ* to obtain the maximum voltage gain with the critical maximum duty cycle conditions is

(41) $$\lambda_{b}=\left(1+\frac{1}{M_{\max}}\right)D_{crit-\max}-1.$$

5. Select the value of *λ* as the lowest between $\lambda_{a}$ and $\lambda_{b}$, i.e.,
  (42) $$\lambda =\min\{\lambda_{a,},\lambda_{b}\},$$

**Figure 5:**
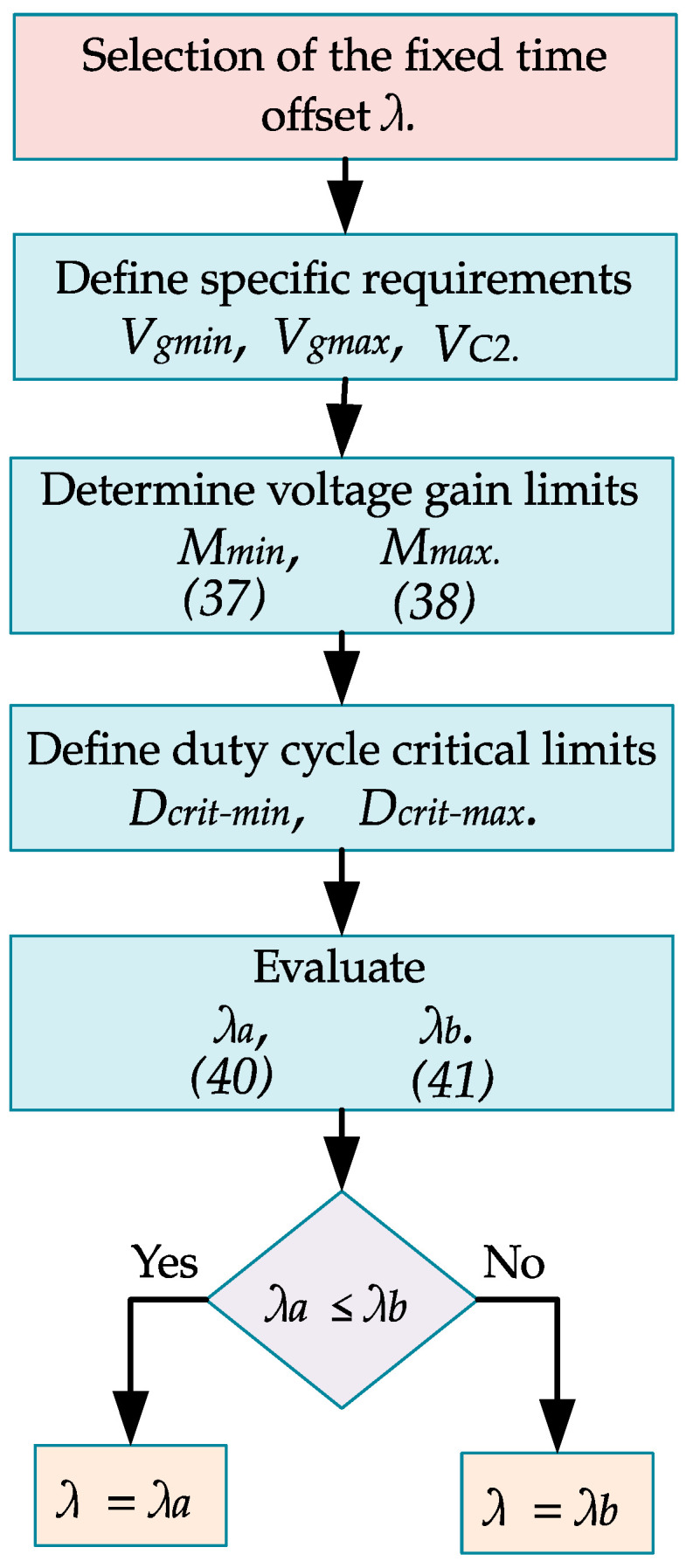
Flowchart illustrating the selection process for the fixed time offset *λ*.

This is the highest *λ* that achieves the set gain value without exceeding the duty cycle critical limits. Figure 5 shows a flowchart of this methodology.

**Figure 6:**
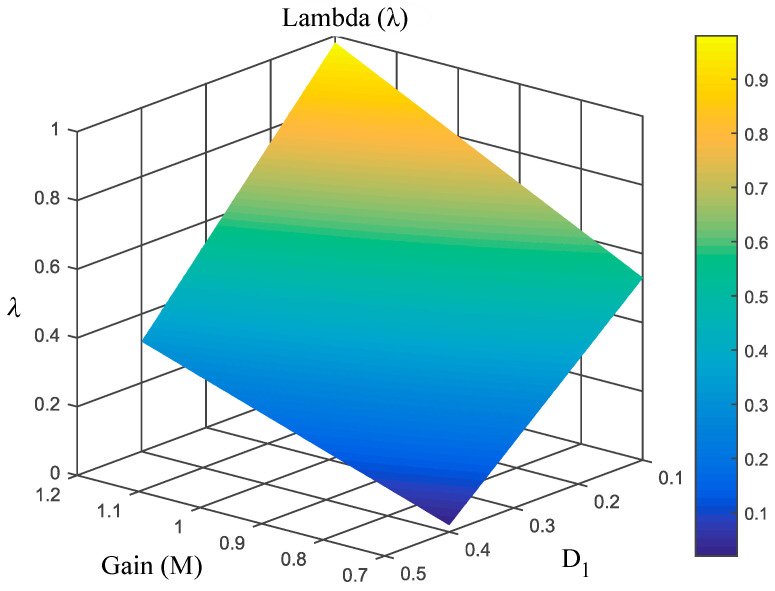
Values of *λ* for a corresponding duty cycle and voltage gain.

Figure 6 illustrates the relationship between λ, duty cycle, and voltage gain, serving as a practical guide for determining the optimal *λ* value according to design specifications. This graphic shows how the nominal duty cycle $D_{1}$ and the range of the voltage gain decrease when *λ* increases. The ripple values obtained in Equations (32)–(35) are directly proportional to $D_{1}$. Thus, the ripple current in inductors and the ripple voltage in capacitors decrease in the same proportion as the nominal duty cycle decreases. According to Equation (27), the average voltage of the transfer capacitor $V_{C1}$ also decreases. This is also beneficial for semiconductors, since according to the Equation (36), the stress voltage $V_{STRESS}$ is reduced significantly as $D_{1}$ decreases.

## 4. Control Design

Following the linearization methods presented in [21], the system model is linearized to design the loop shaping control law. Accordingly, for the system dynamics described by Equations (23)–(26), a linearization process is applied around the equilibrium point, taking into account the steady-state conditions defined in Equations (27)–(30). In this process, the control signal and the four state variables are split into two components referred to as the nominal average values (uppercase letters) and their deviations (letters with a superscript “~”), i.e.,

$$\begin{array}{l} i_{L1}=I_{L1}+{\tilde{i}}_{L1},\\ i_{L2}=I_{L2}+{\tilde{i}}_{L2},\\ v_{C1}=V_{C1}+{\tilde{v}}_{C1},\\ v_{C2}=V_{C2}+{\tilde{v}}_{C2},\\ d_{1}=D_{1}+{\tilde{d}}_{1}. \end{array}$$

Thus, the linearized system around the specified equilibrium point is written as,

(43) $$\dot{\tilde{x}}=A\tilde{x}+B{\tilde{d}}_{1},$$

(44) $$\tilde{y}=C\tilde{x}+E{\tilde{d}}_{1},$$

where $x={\left[i_{L1},i_{L2},v_{C1},v_{C2}\right]}^{T}$, $\tilde{x}={\left[{\tilde{i}}_{L1},{\tilde{i}}_{L2},{\tilde{v}}_{C1},{\tilde{v}}_{C2}\right]}^{T}$ and ${\tilde{d}}_{1}={d_{1}-D}_{1}$. The equilibrium point corresponds to $x_{e}={\left[I_{L1},I_{L2},V_{C1},V_{C2}\right]}^{T}$ and the output vector to $\tilde{y}$. The function $f\left(x,d_{1}\right)={\left[f_{1}\left(x,d_{1}\right),f_{2}\left(x,d_{1}\right),f_{3}\left(x,d_{1}\right),f_{4}\left(x,d_{1}\right)\right]}^{T}={\left[\frac{di_{L1}}{dt},\frac{di_{L2}}{dt},\frac{dv_{C1}}{dt},\frac{dv_{C2}}{dt}\right]}^{T}$ obtained from expressions (23)–(26). Thus, the matrices ***A*** and ***B*** are evaluated as,

(45) $$A={\left[\begin{array}{cccc} \frac{\partial }{\partial i_{L1}}f_{1}(x,d_{1}) & \frac{\partial }{\partial i_{L2}}f_{1}(x,d_{1}) & \frac{\partial }{\partial v_{C1}}f_{1}(x,d_{1}) & \frac{\partial }{\partial v_{C2}}f_{1}(x,d_{1})\\ \frac{\partial }{\partial i_{L1}}f_{2}(x,d_{1}) & \frac{\partial }{\partial i_{L2}}f_{2}(x,d_{1}) & \frac{\partial }{\partial v_{C1}}f_{2}(x,d_{1}) & \frac{\partial }{\partial v_{C2}}f_{2}(x,d_{1})\\ \frac{\partial }{\partial i_{L1}}f_{3}(x,d_{1}) & \frac{\partial }{\partial i_{L2}}f_{3}(x,d_{1}) & \frac{\partial }{\partial v_{C1}}f_{3}(x,d_{1}) & \frac{\partial }{\partial v_{C2}}f_{3}(x,d_{1})\\ \frac{\partial }{\partial i_{L1}}f_{4}(x,d_{1}) & \frac{\partial }{\partial i_{L2}}f_{4}(x,d_{1}) & \frac{\partial }{\partial v_{C1}}f_{4}(x,d_{1}) & \frac{\partial }{\partial v_{C2}}f_{4}(x,d_{1}) \end{array}\right]}_{x_{e},\;D_{1}},$$

(46) $$B={\left[\begin{array}{c} \frac{\partial }{\partial d_{1}}f_{1}(x,d_{1})\\ \frac{\partial }{\partial d_{1}}f_{2}(x,d_{1})\\ \frac{\partial }{\partial d_{1}}f_{3}(x,d_{1})\\ \frac{\partial }{\partial d_{1}}f_{4}(x,d_{1}) \end{array}\right]}_{x_{e},\;D_{1}},$$

where each partial derivative is calculated, neglecting the products of variations corresponding to higher-than-second-order terms in the Taylor series expansion, and evaluated at the equilibrium point $x_{e}$ and the nominal duty cycle $D_{1}.$ For the current-mode controller, the only outputs of the system to sense are the input current $i_{L1}$ and the output voltage $v_{C2}$. Thus, matrices ***C*** and ***E*** are defined as,

(47) $$C=\left[\begin{array}{cccc} 1 & 0 & 0 & 0\\ 0 & 0 & 0 & 0\\ 0 & 0 & 0 & 0\\ 0 & 0 & 0 & 1 \end{array}\right],\;E=\left[\begin{array}{c} 0\\ 0\\ 0\\ 0 \end{array}\right].$$

The resulting linearized model is represented as follows,

(48) $$\left[\begin{array}{c} \begin{array}{l} {\dot{\tilde{i}}}_{L1} \end{array}\\ \begin{array}{l} {\dot{\tilde{i}}}_{L2} \end{array}\\ \begin{array}{l} {\dot{\tilde{v}}}_{C1} \end{array}\\ {\dot{\tilde{v}}}_{C2} \end{array}\right]=\left[\begin{array}{cccc} 0 & 0 & -\frac{1-D_{1}}{L_{1}} & -\frac{1-D_{1}}{L_{1}}\\ 0 & 0 & \frac{D_{1}+\lambda }{L_{2}} & -\frac{1-D_{1}-\lambda }{L_{2}}\\ -\frac{1-D_{1}}{C_{1}} & -\frac{D_{1}+\lambda }{C_{1}} & 0 & 0\\ \frac{1-D_{1}}{C_{2}} & -\frac{1-D_{1}-\lambda }{C_{2}} & 0 & \frac{1}{C_{2}R} \end{array}\right]\left[\begin{array}{c} \begin{array}{l} {\tilde{i}}_{L1} \end{array}\\ \begin{array}{l} {\tilde{i}}_{L2} \end{array}\\ \begin{array}{l} {\tilde{v}}_{C1} \end{array}\\ {\tilde{v}}_{C2} \end{array}\right]+\left[\begin{array}{c} \frac{V_{g}}{(1-D_{1})L_{1}}\\ \frac{V_{g}}{(1-D_{1})L_{2}}\\ -\frac{(1+\lambda )(D_{1}+\lambda )V_{g}}{{\left(1-D_{1}\right)}^{2}RC_{1}}\\ -\frac{(1+\lambda )(D_{1}+\lambda )V_{g}}{{\left(1-D_{1}\right)}^{2}RC_{2}} \end{array}\right]{\tilde{d}}_{1},$$

(49) $$\tilde{y}=\left[\begin{array}{c} {\tilde{i}}_{L1}\\ 0\\ 0\\ {\tilde{v}}_{C2} \end{array}\right].$$

The PI–PI current-mode controller is designed using the input inductor current and output voltage as the primary control variables. Based on the system model described by Equations (48) and (49), Laplace transforms are applied to derive the transfer functions relevant to control system design according to,

(50) $$\frac{\tilde{y}(s)}{d_{1}(s)}=C{\left(sI-A\right)}^{-1}B+E.$$

Thus, the following transfer functions are obtained,

(51) $$\frac{{\tilde{i}}_{L1}(s)}{{\tilde{d}}_{1}(s)}=\frac{b_{3}s^{3}+b_{2}s^{2}+b_{1}s+b_{0}}{s^{4}+a_{3}s^{3}+a_{2}s^{2}+a_{1}s+a_{0}},$$

(52) $$\frac{{\tilde{v}}_{C2}(s)}{{\tilde{d}}_{1}(s)}=\frac{c_{3}s^{3}+c_{2}s^{2}+c_{1}s+c_{0}}{s^{4}+a_{3}s^{3}+a_{2}s^{2}+a_{1}s+a_{0}}.$$

The values of each coefficient are provided in Table 1.

**Figure 7:**
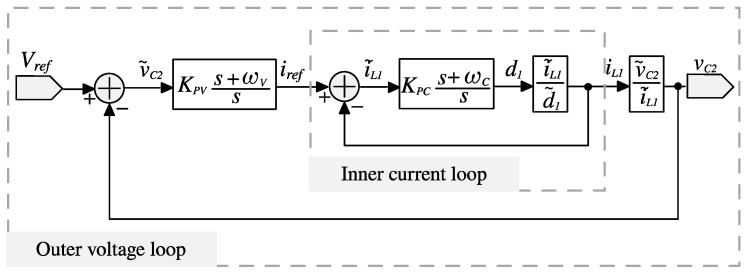
Block diagram of the proposed PI–PI current-mode controller.

After examining both transfer functions, it was observed that all the poles are situated in the left-hand side (LHS) of the s-plane. Additionally, ${\tilde{i}}_{L1}(s)/{\tilde{d}}_{1}(s)$ exhibits minimum phase behavior because its zeros are in the LHS of the s-plane. On the other hand, ${\tilde{v}}_{C2}(s)/{\tilde{d}}_{1}\left(s\right)$ is non-minimum phase since it has zeros in the right-hand side (RHS) of the s-plane. Therefore, a current-mode control strategy based on a loop shaping is proposed to ensure the desired performance of the converter under the improved operating conditions. The proposed PI–PI current-mode controller is shown in Figure 7.

### 4.1. Inner Current Loop

The selection of the controller parameters is based on loop shaping techniques [22]. Therefore, it is necessary to obtain the current loop gain. According to the proposed control scheme shown in Figure 7, the current loop gain is given by,

(53) $$T_{C}=K_{PC}\left(\frac{s+\omega_{C}}{s}\right)\left(\frac{{\tilde{i}}_{L1}}{{\tilde{d}}_{1}}\right).$$

To select the zero $\omega_{C}$, the Bode diagram of the transfer function ${\tilde{i}}_{L1}(s)/{\tilde{d}}_{1}(s)$ is obtained. Then, the zero $\omega_{C}$ is chosen around the half of the resonant frequency. The transfer function ${\tilde{i}}_{L1}(s)/{\tilde{d}}_{1}(s)$ is non-minimum phase. Therefore, a gain $K_{PC}$ is selected such that the crossover frequency of the inner current loop is higher than that of the outer voltage loop, ensuring a faster dynamic response, while remaining below the switching frequency to preserve system stability. Afterward, the Bode diagram of the current loop gain $T_{C}$ is obtained considering $K_{PC}=1$. Subsequently, the proportional gain $K_{PC}$ is adjusted so that the crossover frequency is roughly one decade below the switching frequency with a phase margin greater than 40 degrees. An example of this parameter choice is shown later in Section 5.

### 4.2. Outer Voltage Loop

Since the outer voltage loop exhibits non-minimum phase behavior, the following conditions are satisfied in the loop to ensure robust stability [23]:

1. For relative stability, the slope at the crossover frequency must be approximately −20 dB/dec.
2. To improve steady-state accuracy, the gain at low frequencies should be high.
3. For robust stability, ensure a gain margin greater than 10 dB and a phase margin greater than 60 degrees.

According to the proposed control scheme shown in Figure 7, the outer voltage loop gain yields in

(54) $$T_{V}=K_{PV}\left(\frac{s+\omega_{V}}{s}\right)\left[\frac{K_{PC}\left(\frac{s+\omega_{C}}{s}\right)\left(\frac{{\tilde{i}}_{L1}}{{\tilde{d}}_{1}}\right)}{1+K_{PC}\left(\frac{s+\omega_{C}}{s}\right)\left(\frac{{\tilde{i}}_{L1}}{{\tilde{d}}_{1}}\right)}\right]\left(\frac{{\tilde{v}}_{C2}}{{\tilde{i}}_{L1}}\right).$$

To select the zero $\omega_{V}$, the Bode diagram of the transfer function ${\tilde{v}}_{C2}(s)/{\tilde{d}}_{1}\left(s\right)$ is obtained. Then, the zero $\omega_{V}$ is chosen around the resonant frequency. Afterward, the Bode diagram of the current loop gain $T_{V}$ is obtained considering $K_{PV}=1$. Following this, the proportional gain $K_{PV}$ is adjusted so that the crossover frequency is approximately one decade or more below that of the current loop gain, ensuring a slope of −20 dB/dec and adequate phase and gain margins. An example of this parameter selection is provided in Section 5.

## 5. Results and Comments

In this section, results are presented to assess the performance of the proposed switching strategy. This control strategy is intended for voltage regulation applications within the 200 V to 250 V range, such as systems powered by lithium-ion battery packs, where voltage fluctuations occur according to the battery’s state of charge. Hence, the nominal output voltage is regulated to 220 V, considering this value along with the nominal power required by the load.

The system is powered by a DC source NA8742A, which is used to emulate the system’s behavior under critical operating conditions. The output power is 570 W and the switching frequency of 100 kHz. The converter parameters are listed in Table 2. It is important to note that the voltage and current ratings of the semiconductors used significantly exceed the actual operating conditions observed during experimentation. This is due to two main factors. First, the standard practice is to incorporate a safety margin (typically greater than 1.5) to ensure the protection of both personnel and equipment [24]. Second, the current research was conducted within an academic setting, where the availability of components at the time of implementation is limited, leading to the use of devices with varying voltage and current ratings.

**Figure 8:**
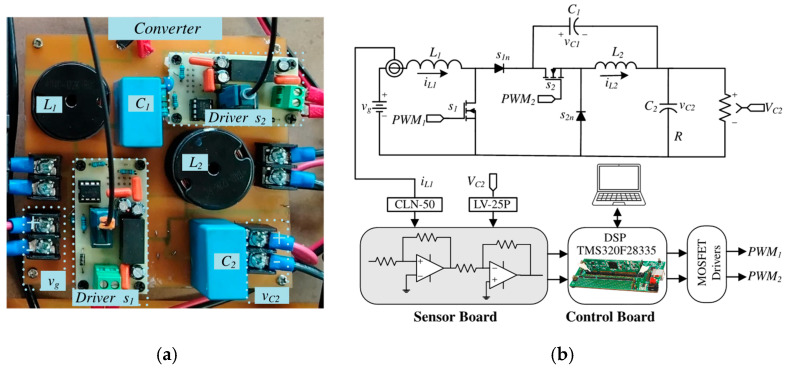

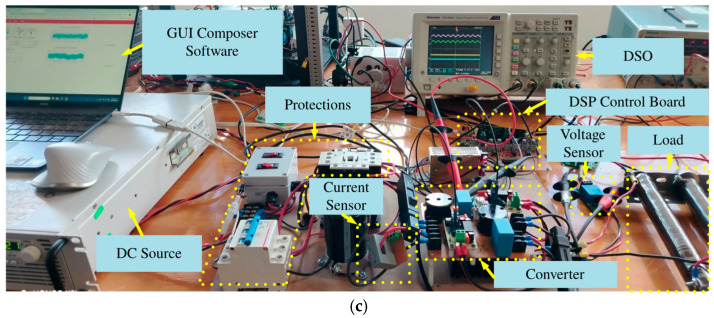
Experimental prototype: (**a**) the proposed converter board, (**b**) block diagram of the closed-loop converter, (**c**) complete experimental setup.

The converter implementation, including key components and layout, is depicted in Figure 8a. The schematic of the experimental setup is presented in Figure 8b, which includes the DSP TMS320F28335 used to implement the proposed control scheme, the signal sensing board, the MOSFET driver stage, and the converter topology. Furthermore, Figure 8c shows the complete experimental setup.

### 5.1. Selection of λ for the Practical Case

To select the optimal possible λ value for the practical case, the methodology described in Section 3 is followed. According to Table 2, the specific voltage requirements are: $V_{min}=200\;V$, $V_{max}=250\;V$, $V_{ref}=V_{C2}=220\;V$. The minimum and maximum voltage gain values are, $M_{min}=220\;V/250\;V=0.88$ and $M_{max}=220\;V/200\;V=1.1$. To accurately determine the critical duty cycles, the voltage gain of the converter must be calculated, taking into account the parasitic effects [25]. Although this analysis falls outside the scope of the present work, it represents a valuable direction for future research. Therefore, for the purpose of demonstrating the proposed operating mode, the minimum and maximum critical duty cycles are selected as $D_{crit-min}=0.2$ and $D_{crit-max}=0.8$. Afterwards, Equations (40) and (41) are evaluated, yielding to $\lambda_{a}=0.5$ and $\lambda_{b}=0.53$. According to Equation (42), the selected value is λ=0.5. This is the highest value of *λ* that ensures achieving the set voltage gain values without exceeding the duty cycle critical limits. For a proper comparison and validation of the proposed mode of operation, experimental results are obtained at different values of λ, i.e., λ=0, λ=0.25, and λ=0.5.

### 5.2. Parameters of the Controller

**Figure 9:**
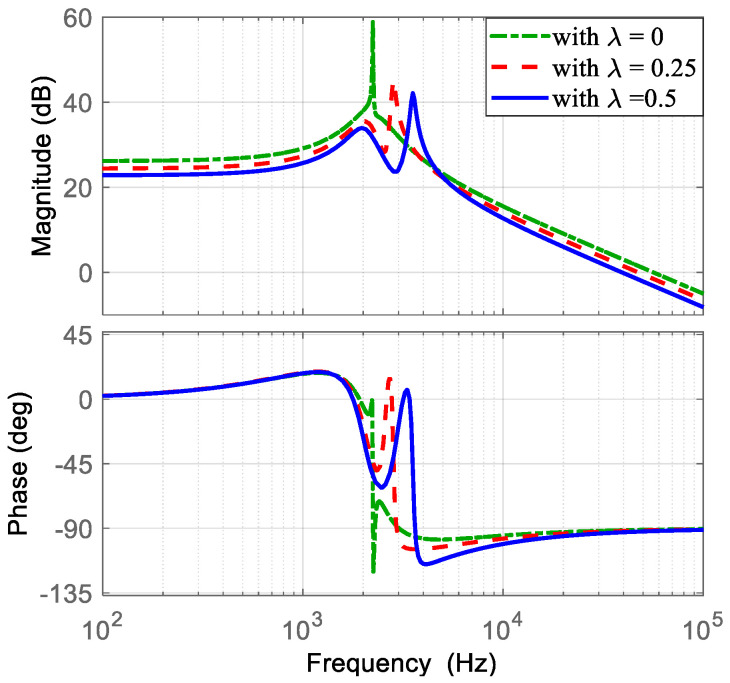
Bode diagram of the transfer function ${\tilde{i}}_{L1}(s)/{\tilde{d}}_{1}(s)$: (**top**) magnitude (*y*-axis: 20 dB/div), and (**bottom**) phase (*y*-axis: 45 deg/div).

**Figure 10:**
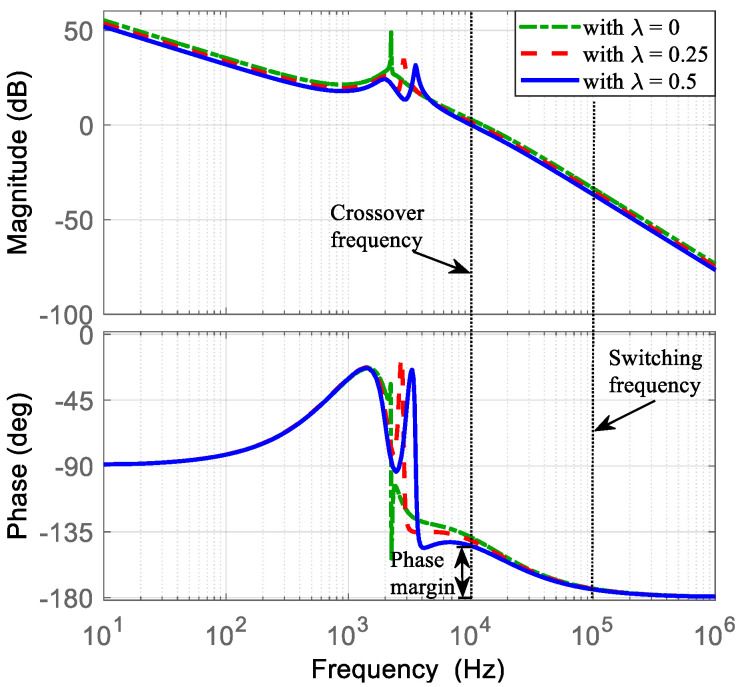
Bode diagram of the current loop gain $T_{C}$ with $K_{PC}$ = 0.003: (**top**) magnitude (*y*-axis: 50 dB/div), and (**bottom**) phase (*y*-axis: 45 deg/div).

A PI-PI current-mode controller is used to regulate the converter output voltage under the proposed operating mode. Section 4 described the methodology for selecting the appropriate controller parameters, and the control scheme was shown in Figure 7. Following this methodology, the current PI controller parameters are chosen. The transfer function ${\tilde{i}}_{L1}(s)/{\tilde{d}}_{1}(s)$, obtained in Equation (51), is evaluated using the converter parameters listed in Table 2 and different values of *λ* (*λ* = 0, *λ* = 0.25, and *λ* = 0.5). Then, the Bode diagrams of this transfer functions are obtained as shown in Figure 9. Here, the location of the zero $\omega_{C}$ is recommended around half of the resonant frequencies. The resonant frequencies appear between 2000 Hz and 4000 Hz. Hence, $\omega_{C}$ is selected at 1500 Hz (9425 rad/s). Subsequently, the current loop gain $T_{C}$ obtained in Equation (53) can be evaluated with a gain of $K_{PC}$ = 1. Afterwards, the Bode diagrams of $T_{C}$ are obtained. Thus, $K_{PC}$ is adjusted such that the crossover frequency is around a decade below of the switching frequency, with adequate phase margin as shown in Figure 10. The selected gain that fulfills the requirements is $K_{PC}$ = 0.3, resulting in a crossover frequency of 10 kHz and a phase margin of 40 degrees.

**Figure 11:**
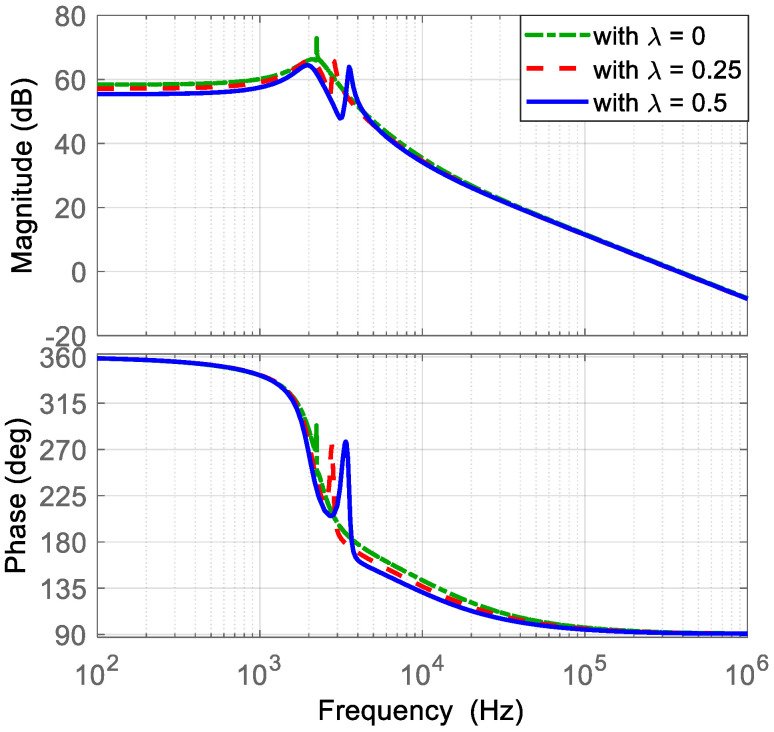
Bode diagram of the transfer function ${\tilde{v}}_{C2}(s)/{\tilde{d}}_{1}(s)$: (**top**) magnitude (*y*-axis: 20 dB/div), and (**bottom**) phase (*y*-axis: 45 deg/div).

**Figure 12:**
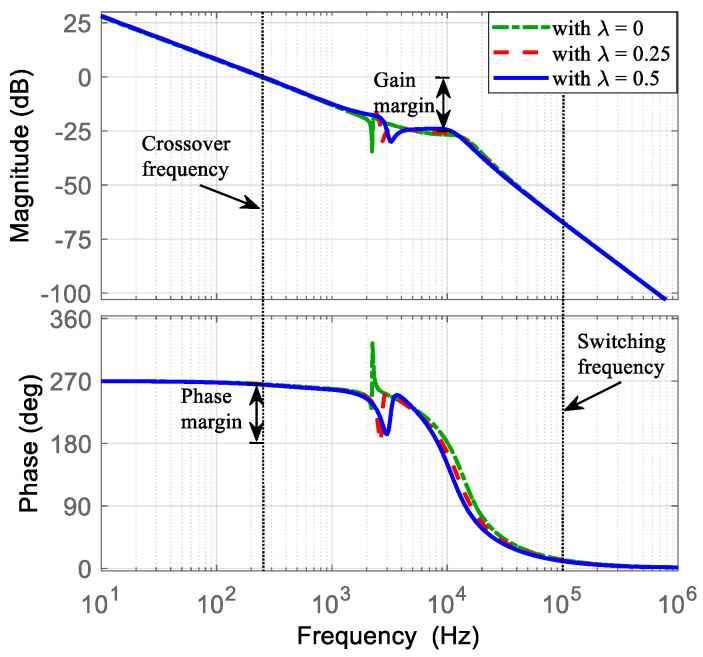
Bode diagram of the current loop gain $T_{V}$ with $K_{PC}$ = 0.003: (**top**) magnitude (*y*-axis: 25 dB/div), and (**bottom**) phase (*y*-axis: 90 deg/div).

At this stage, the parameters of the voltage PI controller are selected. The transfer function ${\tilde{v}}_{C2}(s)/{\tilde{d}}_{1}\left(s\right)$, obtained in Equation (52), is evaluated using the converter parameters listed in Table 2 and considering different values of *λ* (*λ* = 0, *λ* = 0.25, and *λ* = 0.5). Then, the Bode diagrams of this transfer function are obtained as shown in Figure 11. Here, the location of the zero $\omega_{V}$ is recommended around the resonant frequencies. Hence, $\omega_{V}$ is selected at 3000 Hz (18850 rad/s). Subsequently, the current loop gain $T_{V}$ obtained in Equation (54) is evaluated with a gain of $K_{PV}$ = 1. Afterwards, the Bode diagrams of $T_{V}$ are obtained. Thus, $K_{PV}$ is adjusted in such a way that the slope in the crossover frequency is −20 dB/dec and is a decade lower than the crossover frequency, at least, of the current loop gain, with an adequate gain and phase margin as shown in Figure 12. The selected gain that fulfills the requirements is $K_{PC}$ = 0.003, resulting in a crossover frequency of 10 kHz, a gain margin of 22 dB, and a phase margin of 86 degrees.

After the selection of the controller parameters. The PI–PI current-mode controller is implemented in the prototype. To assess the performance of the converter in a closed-loop, four different sets of tests are considered, steady-state validations, stepwise changes in the load, changes in the input voltage, and stepwise changes in the voltage reference.

### 5.3. Steady-State Validations

**Figure 13:**
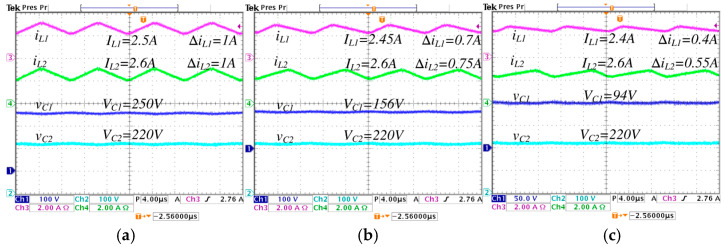
Steady-state responses of the converter operating in step-down mode, regulating the input voltage $V_{g}=250\;V$ to the output voltage $V_{C2}=220\;V$, with an output power $P_{out}=570\;W$, for different values of *λ*: (**a**) *λ* = 0, (**b**) *λ* = 0.25, and (**c**) *λ* = 0.5. (From top to bottom) inductor current $i_{L1}$ (*y*-axis: 2 A/div), inductor current $i_{L2}$ (*y*-axis: 2 A/div), capacitor voltage $v_{C1}$ (*y*-axis: 100 V/div), and capacitor voltage $v_{C2}$ (*y*-axis: 100 V/div) (*x*-axis: time 4 μs/div).

**Figure 14:**
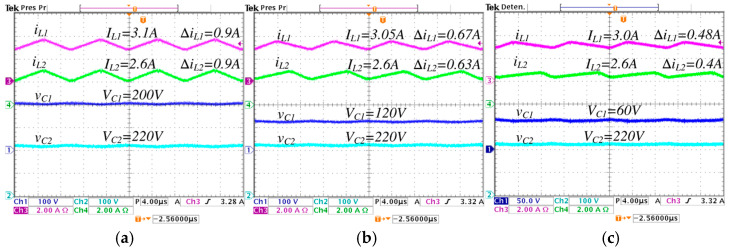
Steady-state responses of the converter operating in step-up mode, regulating the input voltage $V_{g}=200\;V$ to the output voltage $V_{C2}=220\;V$, with an output power $P_{out}=570\;W$, for different values of *λ*: (**a**) *λ* = 0, (**b**) *λ* = 0.25, and (**c**) *λ* = 0.5. (From top to bottom) inductor current $i_{L1}$ (*y*-axis: 2 A/div), inductor current $i_{L2}$ (*y*-axis: 2 A/div), capacitor voltage $v_{C1}$ (*y*-axis: 100 V/div), and capacitor voltage $v_{C2}$ (*y*-axis: 100 V/div) (*x*-axis: time 4 μs/div).

To validate the proposed improved operation of the converter, experimental tests are conducted on different values of λ, i.e., λ=0, λ=0.25, and λ=0.5. Where λ=0 corresponds to conventional synchronized switching, and λ=0.5 is the highest value of *λ* that ensures achieving the set voltage gain values without exceeding the duty cycle critical limits. The aim is to compare the results and highlight the advantages of the proposed improved operation. Figure 13 and Figure 14 show the steady state response of the state variables considering an output voltage of 570 W and a voltage reference $V_{ref}=220$ V.

In Figure 13, the converter operates in step-down mode, while in Figure 14 operates in step-up mode. In Figure 13, a comparison is performed under different values of λ from 0 to 0.5, the following is observed: the average inductor current $I_{L1}$ (input current) decreases from 2.5 A to 2.4 A, and the ripple ${∆i}_{L1}$ decreases from 1 A to 0.4 A; the average inductor current $I_{L2}$ is maintained at 2.6 A, and the ripple ${∆i}_{L2}$ decreases from 1 A to 0.55 A; the average capacitor voltage $V_{C1}$ significantly decreases from 250 V to 94 V; and the average capacitor voltage $V_{C2}$ (output voltage) is maintained constant at 220 V.

In Figure 14, a comparison is performed under different values of λ from 0 to 0.5, the following is observed: the average inductor current $I_{L1}$ decreases from 3.1 A to 3.0 A, and the ripple ${∆i}_{L1}$ decreases from 0.9 A to 0.48 A; the average inductor current $I_{L2}$ is maintained at 2.6 A, and the ripple ${∆i}_{L2}$ decreases from 0.9 A to 0.4 A; the average capacitor voltage $V_{C1}$ significantly decreases from 200 V to 60 V; and the average capacitor voltage $V_{C2}$ is maintained constant at 220 V.

**Figure 15:**
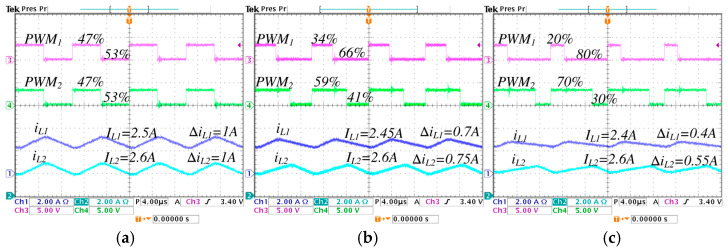
Inductor current ripples and pulse-width modulations of the converter operating in step-down mode, regulating the input voltage $V_{g}=250\;V$ to the output voltage $V_{C2}=220\;V$, with an output power $P_{out}=570\;W$, for different values of *λ*: (**a**) *λ* = 0, (**b**) *λ* = 0.25, and (**c**) *λ* = 0.5. (From top to bottom) pulse-width modulation of the first active switch ${PWM}_{1}$ (*y*-axis: 5 V/div), pulse-width modulation of the second active switch ${PWM}_{2}$ (*y*-axis: 5 V/div), inductor current $i_{L1}$ (*y*-axis: 2 A/div), and inductor current $i_{L2}$ (*y*-axis: 2 A/div) (*x*-axis: time 4 μs/div).

**Figure 16:**
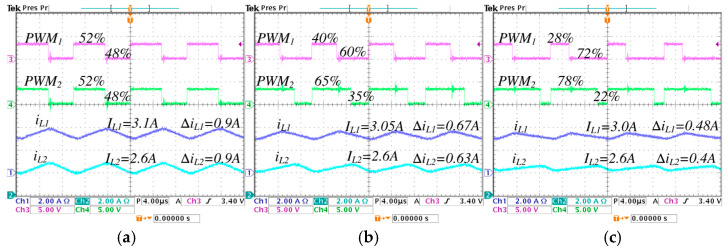
Inductor current ripples and pulse-width modulations of the converter operating in step-up mode, regulating the input voltage $V_{g}=200\;V$ to the output voltage $V_{C2}=220\;V$, with an output power $P_{out}=570\;W$, for different values of *λ*: (**a**) *λ* = 0, (**b**) *λ* = 0.25, and (**c**) *λ* = 0.5. (From top to bottom) pulse-width modulation of the first active switch ${PWM}_{1}$ (*y*-axis: 5 V/div), pulse-width modulation of the second active switch ${PWM}_{2}$ (*y*-axis: 5 V/div), inductor current $i_{L1}$ (*y*-axis: 2 A/div), and inductor current $i_{L2}$ (*y*-axis: 2 A/div) (*x*-axis: time 4 μs/div).

Figure 15 and Figure 16 show the inductor current ripples and the pulse-width modulations of the converter. In Figure 15, the converter operates in step-down mode, while in Figure 16 operates in step-up mode. In Figure 15, a comparison is performed under different values of λ from 0 to 0.5, the following is observed: the pulse-width modulation of the active switch $s_{1}$, represented by ${PWM}_{1}$, change from 47% to 20%; the pulse-width modulation of the active switch $s_{2}$, represented by ${PWM}_{2}$, change from 47% to 70%; the average inductor current $I_{L1}$ decreases from 2.5 A to 2.4 A, and the ripple ${∆i}_{L1}$ decreases from 1 A to 0.4 A; the average inductor current $I_{L2}$ is maintained at 2.6 A, and the ripple ${∆i}_{L2}$ decreases from 1 A to 0.55 A.

In Figure 16, a comparison is performed under different values of λ from 0 to 0.5, the following is observed: the pulse-width modulation of the active switch $s_{1}$, represented by ${PWM}_{1}$, change from 52% to 28%; the pulse-width modulation of the active switch $s_{2}$, represented by ${PWM}_{2}$, change from 52% to 78%; the average inductor current $I_{L1}$ decreases from 3.1 A to 3 A, and the ripple ${∆i}_{L1}$ decreases from 0.9 A to 0.48 A; the average inductor current $I_{L2}$ is maintained at 2.6 A, and the ripple ${∆i}_{L2}$ decreases from 0.9 A to 0.4 A.

The minimum pulse is presented in ${PWM}_{1}$, when the converter works in step-down operation mode with *λ* = 0.5, equivalent to a duty cycle of 0.2 (20%), as seen in Figure 15c. The maximum pulse is presented in ${PWM}_{2}$, when the converter works in step-up operation mode with *λ* = 0.5, equivalent to a duty cycle of 0.78 (78%), as seen in Figure 16c. It is important to note that the established critical duty cycle limits are not exceeded.

**Figure 17:**
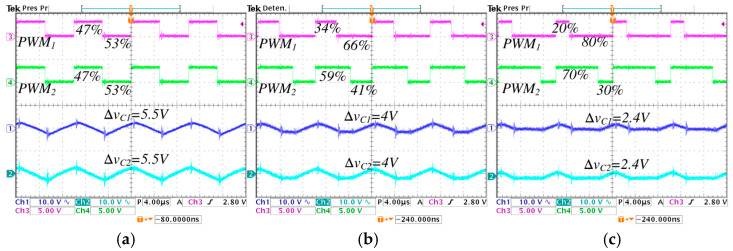
Capacitor voltage ripples and pulse-width modulations of the converter operating in step-down mode, regulating the input voltage $V_{g}=250\;V$ to the output voltage $V_{C2}=220\;V$, with an output power $P_{out}=570\;W$, for different values of *λ*: (**a**) *λ* = 0, (**b**) *λ* = 0.25, and (**c**) *λ* = 0.5. (From top to bottom) pulse-width modulation of the first active switch ${PWM}_{1}$ (*y*-axis: 5 V/div), pulse-width modulation of the second active switch ${PWM}_{2}$ (*y*-axis: 5 V/div), capacitor voltage ripple $\Delta v_{C1}$ (*y*-axis: 5 V/div), and capacitor voltage ripple $\Delta v_{C2}$ (*y*-axis: 5 V/div) (*x*-axis: time 4 μs/div).

**Figure 18:**
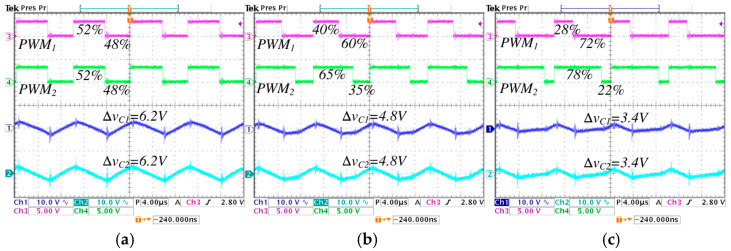
Capacitor voltage ripples and pulse-width modulations of the converter operating in step-up mode, regulating the input voltage $V_{g}=200\;V$ to the output voltage $V_{C2}=220\;V$, with an output power $P_{out}=570\;W$, for different values of *λ*: (**a**) *λ* = 0, (**b**) *λ* = 0.25, and (**c**) *λ* = 0.5. (From top to bottom) pulse-width modulation of the first active switch ${PWM}_{1}$ (*y*-axis: 5 V/div), pulse-width modulation of the second active switch ${PWM}_{2}$ (*y*-axis: 5 V/div), capacitor voltage ripple $\Delta v_{C1}$ (*y*-axis: 5 V/div), and capacitor voltage ripple $\Delta v_{C2}$ (*y*-axis: 5 V/div) (*x*-axis: time 4 μs/div).

Figure 17 and Figure 18 show the form of capacitor voltage ripples and the pulse-width modulations of the converter. In Figure 17 the converter operates in step-down mode, while in Figure 18 operates in step-up mode. In Figure 17, a comparison is performed under different values of λ from 0 to 0.5, the following is observed: the pulse-width modulation of the active switch $s_{1}$, represented by ${PWM}_{1}$, change from 47% to 20%; the pulse-width modulation of the active switch $s_{2}$, represented by ${PWM}_{2}$, change from 47% to 70%; the voltage ripple ${∆v}_{C1}$ and ${∆v}_{C2}$ decrease from 5.5 V A to 2.4 V. In Figure 18, a comparison is performed under different values of *λ* from 0 to 0.5, the following is observed: the pulse-width modulation of the active switch $s_{1}$, represented by ${PWM}_{1}$, change from 52% to 28%; the pulse-width modulation of the active switch $s_{2}$, represented by ${PWM}_{2}$, change from 52% to 78%; the voltage ripple ${∆v}_{C1}$ and ${∆v}_{C2}$ decrease from 6.2 V A to 3.4 V.

All these steady-state graphics demonstrate that the converter is capable of both stepping up and stepping down the voltage, reducing current and voltage ripple. They also validate the expressions for the average values of the state variables, the ripple expressions, and the voltage gain expression obtained in Section 3 with the proposed asynchronous operation.

**Figure 19:**
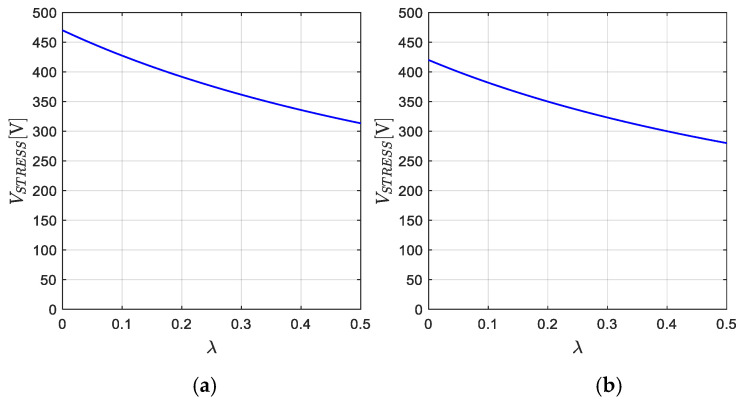
Stress voltage on semiconductors with respect *λ* when the converter: (**a**) step-down operation mode, the voltage from 250 V to 220 V, (**b**) step-up operation mode, the voltage from 200 V to 220 V.

Another benefit is that the stress voltage of the semiconductor elements is reduced when *λ* increases according to Expression (36). Figure 19 shows the voltage stress curves corresponding to the proposed application. When the converter operates in step-down mode, reducing the input voltage from 250 V to 220 V, the voltage stress on the semiconductors reaches 472 V for *λ* = 0 and decreases to 312 V for *λ* = 0.5. In contrast, during step-up operation from 200 V to 220 V, the voltage stress is 420 V for *λ* = 0 and reduces to 280 V for *λ* = 0.5. All values obtained from the different steady-state tests are summarized in Table 3.

### 5.4. Stepwise Changes in the Load

**Figure 20:**
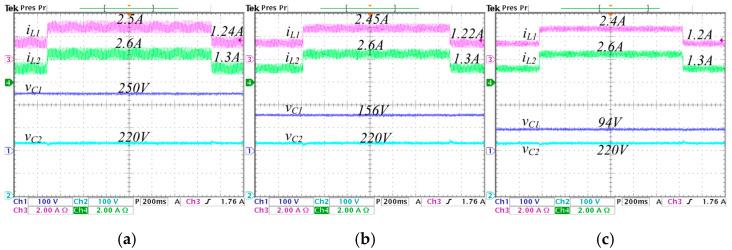
Experimental stepwise changes in the load from 570 W to 285 W with the converter operating in step-down mode, regulating the input voltage $V_{g}=250\;V$ to the output voltage $V_{C2}=220\;V$, for different values of *λ*: (**a**) *λ* = 0, (**b**) *λ* = 0.25, and (**c**) *λ* = 0.5. (From top to bottom) inductor current $i_{L1}$ (*y*-axis: 2 A/div), inductor current $i_{L2}$ (*y*-axis: 2 A/div), capacitor voltage $v_{C1}$ (*y*-axis: 100 V/div), and capacitor voltage $v_{C2}$ (*y*-axis: 100 V/div) (*x*-axis: time 4 μs/div).

**Figure 21:**
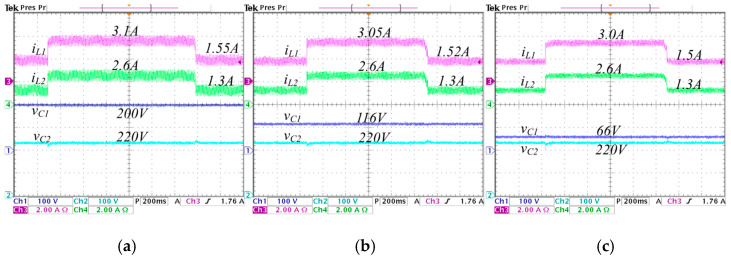
Experimental stepwise changes in the load from 570 W to 285 W with the converter operating in step-up mode, regulating the input voltage $V_{g}=200\;V$ to the output voltage $V_{C2}=220\;V$, for different values of *λ*: (**a**) *λ* = 0, (**b**) *λ* = 0.25, and (**c**) *λ* = 0.5. (From top to bottom) inductor current $i_{L1}$ (*y*-axis: 2 A/div), inductor current $i_{L2}$ (*y*-axis: 2 A/div), capacitor voltage $v_{C1}$ (*y*-axis: 100 V/div), and capacitor voltage $v_{C2}$ (*y*-axis: 100 V/div) (*x*-axis: time 4 μs/div).

To assess the transient performance of the converter under the proposed control strategy, step changes in the load are applied. The load transitions from 570 W to 285 W, corresponding to resistance values of 85 Ω and 170 Ω, respectively, while maintaining a constant voltage reference $V_{ref}=220\;V$. Experimental results under these conditions are shown in Figure 20 and Figure 21. In Figure 20 the converter step-down the voltage from 250 V to 220 V. Here, the current $i_{L1}$ change from 1.24 A to 2.5 A when λ=0, from 1.22 A to 2.45 A when λ=0.25, and from 1.2 A to 2.4 A when λ=0.5; the current $i_{L2}$ change from 1.3 A to 2.6 A in the three λ cases; the voltage $v_{C1}$ is 250 V when λ=0, 156 V when λ=0.25, and 94 V when λ=0.5; the output voltage $v_{C2}$ is 220 V in the three λ cases.

In Figure 21 the converter step-up the voltage from 200 V to 220 V. Here, the current $i_{L1}$ change from 1.55 A to 3.1 A when λ=0, from 1.52 A to 3.05 A when λ=0.25, and from 1.3 A to 1.5 A when λ=0.5; the current $i_{L2}$ change from 1.3 A to 2.6 A in the three λ cases; the voltage $v_{C1}$ is 200 V when λ=0, 116 V when λ=0.25, and 66 V when λ=0.5; the output voltage $v_{C2}$ is 220 V in the three λ cases. Note that in all graphics of both figures, the control action allows the output voltage to remain regulated to the constant reference voltage despite changes in load.

### 5.5. Changes in the Input Voltage

**Figure 22:**
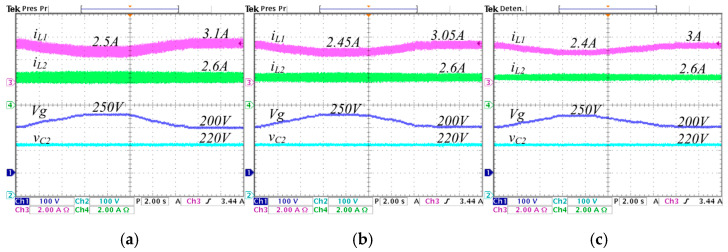
Experimental changes in the input voltage from 200 V to 250 V with the converter regulating to an output voltage of $V_{C2}=220\;V$, and a load of R=85 Ω, for different values of *λ*: (**a**) *λ* = 0, (**b**) *λ* = 0.25, and (**c**) *λ* = 0.5. (From top to bottom) inductor current $i_{L1}$ (*y*-axis: 2 A/div), inductor current $i_{L2}$ (*y*-axis: 2 A/div), input voltage $V_{g}$ (*y*-axis: 100 V/div), and capacitor voltage $v_{C2}$ (*y*-axis: 100 V/div) (*x*-axis: time 2 s/div).

The next test is a change the input voltage from 200 V to 250 V, with the voltage reference fixed at $V_{ref}$ = 220 V at 570 W. The experimental graphics are shown in Figure 22. Here, the current $i_{L1}$ change from 2.5 A to 3.1 A when λ=0, from 2.45 A to 3.05 A when λ=0.25, and from 2.4 A to 3 A when λ=0.5; the current $i_{L2}$ is maintained at 2.6 A in the three λ cases; the voltage $V_{g}$ changes continuously from 200 V to 250 V in the three λ cases; and the output voltage $v_{C2}$ is 220 V in the three λ cases. In all cases, the controller properly maintains the output voltage regulated to the reference voltage despite changes in the input voltage.

### 5.6. Stepwise Changes in the Voltage Reference

**Figure 23:**
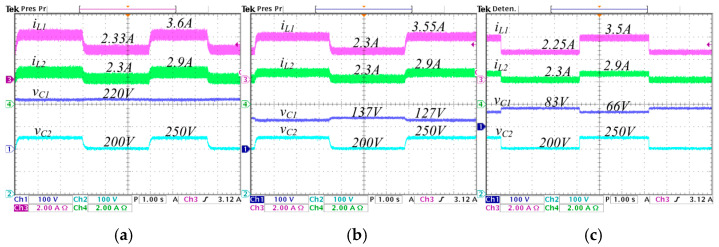
Experimental stepwise changes in the voltage reference from 200 V to 250 V of the converter regulating with an input voltage of $V_{g}=220\;V$ and a load of R = 85 Ω, for different values of *λ*: (**a**) *λ* = 0, (**b**) *λ* = 0.25, and (**c**) *λ* = 0.5. (From top to bottom) input voltage $V_{g}$(*y*-axis: 100 V/div), the output voltage $v_{C2}$ with *λ* = 0.5 (*y*-axis: 100 V/div), the output voltage $v_{C2}$ with *λ* = 0.25 (*y*-axis: 100 V/div), and the output voltage $v_{C2}$ with *λ* = 0 (*y*-axis: 100 V/div) (*x*-axis: time 1 s/div).

The final test involves varying the voltage reference from 200 V to 250 V, with an input voltage $V_{g}$ = 220 V and an output load R = 85 Ω. The corresponding experimental results are presented in Figure 23, which displays, from top to bottom: the input current $i_{L1}$ changing from 2.33 A to 3.6 A when λ=0, from 2.3 A to 3.55 A when λ=0.25, and from 2.25 A to 3.5 A when λ=0.5; the inductor current $i_{L2}$ changing from 2.3 A to 2.9 A at the three λ cases; the capacitor voltage $v_{C1}$ with average value of 220 V when *λ* = 0, 127 V to 137 V when *λ* = 0.25, and 66 V to 83 V when *λ* = 0.5; finally, the output voltage $v_{C2}$ changing from 200 V to 250 V at the three λ cases. In all cases, the output voltage properly follows the changes in the voltage reference, showing an adequate performance of the controller. The dynamic responses are similar at the different values of *λ*, but it can be noted that the ripple current values and the voltage value $v_{C1}$ are reduced as *λ* increases.

**Figure 24:**
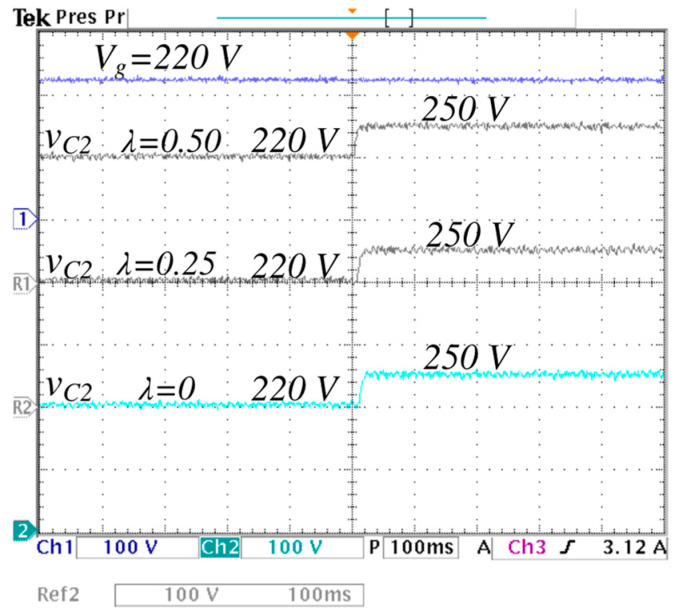
Transient response of the output voltage to stepwise changes in the voltage reference from 200 V to 250 V with an input voltage of $V_{g}=220\;V$ and a load of R = 85 Ω. (From top to bottom) input voltage $V_{g}$ (*y*-axis: 100 V/div), the output voltage $v_{C2}$ with *λ* = 0.5 (*y*-axis: 100 V/div), the output voltage $v_{C2}$ with *λ* = 0.25 (*y*-axis: 100 V/div), and the output voltage $v_{C2}$ with *λ* = 0 (*y*-axis: 100 V/div) (*x*-axis: time 100 ms/div).

To observe the transient response of the output voltage for different values of *λ*, the reference voltage is varied in steps from 200 V to 250 V, as shown in Figure 24. The output voltage properly follows changes in the reference. The settling time for each case is observed to be less than 10 ms and decreases as the lambda increases. It is also noticeable that the response is smooth, without overshoot.

### 5.7. Efficiency Analysis

**Figure 25:**
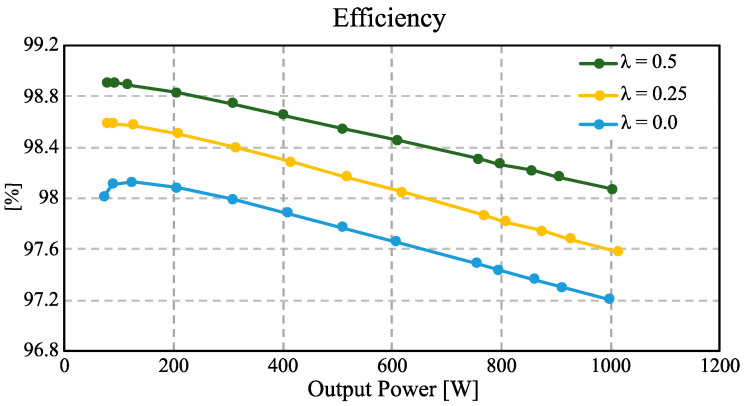
Efficiency of the proposed converter for three different values of *λ*: 0, 0.25, and 0.5.

This subsection presents an efficiency analysis conducted using the thermal module of the PSIM software (Version 9.1). The specific part numbers of the semiconductor devices and other components used in the simulation are listed in Table 2. Parasitic resistances associated with both semiconductor and passive elements were included, with values extracted from manufacturer datasheets. The efficiency was calculated using the following expression:

(55) $$\eta =\frac{P_{out}}{P_{out}+P_{T,lss}},$$

where $P_{T,lss}$ denotes the total power losses, and $P_{out}$ is the output power of the converter. The efficiency is evaluated over an output power range from 70 W to 1 kW. The efficiency analysis was conducted for three different values of *λ*, namely 0, 0.25, and 0.5. As observed in Figure 25, the converter achieves its maximum efficiency when operating with *λ* = 0.5 reaching a peak value of 98.9% at an output power of 80 W.

As illustrated in Figure 19, increasing the values of *λ* contributes to reducing the electrical stress on semiconductor devices, in addition to reducing the current and voltage ripple in passive components such as inductors and capacitors. This reduction in power losses contributes to higher efficiency values compared to when the converter operates with lower values of *λ*, since as the value of *λ* decreases, the electrical stress on semiconductors and the voltage and current ripple in capacitors and inductors increase; consequently, there is an increase in power losses. However, even if the converter operates with a small *λ*, the converter can guarantee efficiencies greater than 97% as observed in Figure 25. This behavior clearly illustrates the impact of *λ* on the converter’s performance, highlighting its suitability for applications in renewable energy systems, battery charging, and energy storage, where maintaining high efficiency and reducing component stress are crucial for system reliability.

### 5.8. Comparison with Other Converters

In this subsection, the proposed converter is compared against other used topologies for similar applications as shown in Table 4. The comparison focuses in terms of the number of components, voltage gain, power efficiency, output power and switching frequency. It is important to highlight that the proposed converter is assessed under higher load conditions compared to the other topologies presented. This distinction emphasizes its robustness and suitability for demanding applications. Furthermore, the proposed converter exhibits higher power efficiency compared to the other topologies analyzed.

## 6. Conclusions

This article presents an enhanced operational strategy for the MNI-SDU DC-DC converter. The proposed approach aims to achieve asynchronous switching of the semiconductors to reduce voltage and current ripples without the need for additional inductors or capacitors. Furthermore, an average model of the system operating in asynchronous mode was developed. Based on this model, a linearization around the equilibrium point was performed to design a loop-shaping control law. The proposed strategy was tested for output voltage regulation in a system that emulates critical behavior typical of battery-powered applications. The system was evaluated under various conditions, including steady-state operation, dynamic load steps, and both step-up and step-down modes.

Experimental results demonstrated the satisfactory performance of the converter. The asynchronous switching strategy contributed to greater flexibility in power distribution and effective ripple shaping, validating the proposed approach. A key element of this strategy is the selection of the duty cycle for each switching element, defined as $d_{2}=d_{1}+\lambda$. This condition ensures asynchronous operation of the converter, significantly reducing current and voltage ripples across inductors and capacitors. Notably, this operating mode enables the converter to either minimize ripple or utilize smaller passive components, thereby improving overall system efficiency and compactness.

A methodology for designing a PI-PI current-mode controller based on loop shaping techniques was described. The controller was implemented in the converter prototype. This regulator underwent different tests, demonstrating adequate voltage regulation despite changes in load, changes in input voltage, and changes in reference voltage. Notably, the proposed control law is capable of operating in both synchronous and asynchronous modes. This flexibility is achieved because the average system model incorporates the relationship between $d_{1}$ and $d_{2}$, effectively generalizing the model to support both switching strategies. It is important to note that all experimental tests were conducted with different values of λ, where λ=0 corresponds to the synchronous operation of the converter.

Experimental results confirmed that this approach leads to significant improvements. When compared to the traditional operation, the proposed method reduced the ripple values by up to 60% and the voltage stress on semiconductors by up to 33%.

## Figures and Tables

**Table 1:** Coefficients of the transfer functions (51) and (52).

Coefficient	Value
$a_{o}$	$\frac{{\left(1-D_{1}\right)}^{2}}{C_{1}C_{2}L_{1}L_{2}}$
$a_{1}$	$\frac{L_{2}{\left(1-D_{1}\right)}^{2}+{\left(D_{1}+\lambda \right)}^{2}L_{1}}{C_{1}C_{2}L_{1}L_{2}R}$
$a_{2}$	$\frac{{\left(1-D_{1}\right)}^{2}(C_{1}L_{1}+C_{1}L_{2}+C_{2}L_{2})+{\left(D_{1}+\lambda \right)}^{2}C_{2}L_{1}+C_{1}L_{1}\lambda \left[\lambda +2(1-D_{1})\right]}{C_{1}C_{2}L_{1}L_{2}}$
$a_{3}$	$\frac{1}{C_{2}R}$
$b_{o}$	$\frac{2V_{g}(1+\lambda )(D_{1}+\lambda )}{C_{1}C_{2}L_{1}L_{2}R(1-D_{1})}$
$b_{1}$	$\frac{V_{g}L_{2}\left[\left(1+\lambda )(D_{1}+\lambda \right)\right]+V_{g}R^{2}\left[C_{1}\lambda (D_{1}+\lambda -1)+C_{2}(1+\lambda )(D_{1}+\lambda )\right]}{C_{1}C_{2}L_{1}L_{2}R^{2}(1-D_{1})}$
$b_{2}$	$\frac{V_{g}\left[C_{1}+(C_{1}+C_{2})(1+\lambda )(D_{1}+\lambda )\right]}{C_{1}C_{2}L_{1}R(1-D_{1})}$
$b_{3}$	$\frac{V_{g}}{L_{1}(1-D_{1})}$
$c_{o}$	$\frac{V_{g}(1+\lambda )}{C_{1}C_{2}L_{1}L_{2}}$
$c_{1}$	$\frac{-V_{g}{D_{1}}^{2}}{C_{1}C_{2}L_{2}R{\left(1-D_{1}\right)}^{2}}$
$c_{2}$	$\frac{V_{g}(L_{1}+L_{2})}{C_{2}L_{1}L_{2}}$
$c_{3}$	$\frac{-V_{g}D_{1}}{C_{2}R{\left(1-D_{1}\right)}^{2}}$

**Table 2:** Parameters of the converter.

Parameter	Value	Part Number
Input voltage, *V_g_*	220V nominal (200–250 V)	---
Reference voltage, *V_ref_*	220 V	---
Switching frequency, *f_S_*	100 kHz	---
$\mathrm{Output}\;\mathrm{power},\;P_{out}$	570 W	---
Load, *R*	75 Ω	---
$\mathrm{Inductor},\;L_{1}$	1.2 mH	1140–122 K
$\mathrm{Inductor},\;L_{2}$	1.2 mH	1140–122 K
$\mathrm{Capacitor},\;C_{1}$	2.2 µF	B32923C3225M000
$\mathrm{Capacitor},\;C_{2}$	2.2 µF	B32923C3225M000
$\mathrm{MOSFET},\;s_{1}$	1200 V, 17 A	IPP026NIONF25
$\mathrm{MOSFET},\;s_{2}$	650 V, 21 A	SCT3120ALGC11
$\mathrm{DIODE},\;s_{1n}$	650 V, 15 A	SCS315AHGC9
$\mathrm{DIODE},\;s_{2n}$	650 V, 15 A	SCS315AHGC9

**Table 3:** Summary of Converter Parameters in Steady-State for Step-Down and Step-Up Modes.

	Step-Down Mode Tests Results			Step-Up Mode Tests Results		
	$V_{g}=250\;V,$ $V_{ref}=220$ V $P_{out}=570\;W$			$V_{g}=200\;V,$ $V_{ref}=220\;V$ $P_{out}=570\;W$		
	*λ* = 0	*λ* = 0.25	*λ* = 0.5	*λ* = 0	*λ* = 0.25	*λ* = 0.5
$D_{1}$	0.47	0.34	0.2	0.52	0.40	0.28
$D_{1}+$ *λ*	0.47	0.59	0.7	0.52	0.65	0.78
$I_{L1}$	2.5 A	2.45 A	2.4 A	3.1 A	3.05 A	3.0 A
$I_{L2}$	2.6 A	2.6 A	2.6 A	2.6 A	2.6 A	2.6 A
$V_{C1}$	250 V	156 V	94 V	200 V	120	60
$V_{C2}$	220 V	220 V	220 V	220 V	220 V	220 V
$\Delta i_{L1}$	1 A	0.7 A	0.4 A	0.9 A	0.67 A	0.48 A
$\Delta i_{L2}$	1 A	0.75 A	0.55 A	1 A	0.63 A	0.4 A
$\Delta v_{C1}$	5.5 V	4 V	2.4 V	6.2 V	4.8 V	3.4 V
$\Delta v_{C2}$	5.5 V	4 V	2.4 V	6.2 V	4.8 V	3.4 V
$V_{STRESS}$	472 V	376 V	312 V	420 V	336 V	280 V

**Table 4:** Comparison between proposed and other converters.

Converter	Proposed λ=0.5	[17] *n* = 2	[18]	[19]	[20]
Switches	2	2	4	1	2
Diodes	2	2	0	1	2
Inductors	2	2	1	1	2
Capacitors	2	1	1	1	2
Voltage gain	$\frac{D+0.5}{1-D}$	$\frac{2D}{1-D}$	$\frac{D}{1-D}$	$\frac{D}{1-D}$	$\frac{D}{{\left(1-D\right)}^{2}}$
Efficiency	98.5%	---	96%	86.7%	91.4%
$P_{out}$	570 W	19.2 W	1.3 W	45 W	247 W
$f_{S}$	100 kHz	20 kHz	700 kHz	50 kHz	50 kHz

## Data Availability

The original contributions presented in this study are included in the article. Further inquiries can be directed to the corresponding authors.
